# Dysbiosis and Implication of the Gut Microbiota in Diabetic Retinopathy

**DOI:** 10.3389/fcimb.2021.646348

**Published:** 2021-03-19

**Authors:** Yinhua Huang, Zhijie Wang, Hongjie Ma, Shangli Ji, Zhongping Chen, Zekai Cui, Jiansu Chen, Shibo Tang

**Affiliations:** ^1^ Aier School of Ophthalmology, Central South University, Changsha, China; ^2^ Aier Eye Institute, Changsha, China; ^3^ Changsha Aier Eye Hospital, Changsha, China; ^4^ Key Laboratory for Regenerative Medicine, Ministry of Education, Jinan University, Guangzhou, China; ^5^ Institute of Ophthalmology, Medical College, Jinan University, Guangzhou, China; ^6^ CAS Center for Excellence in Brain Science and Intelligence Technology, Chinese Academy of Sciences, Shanghai, China

**Keywords:** gut microbiota, human, diabetes mellitus, diabetic retinopathy, 16S rRNA gene sequence

## Abstract

The pathogenesis of type 2 diabetes mellitus (T2DM) is commonly associated with altered gut bacteria. However, whether the microbial dysbiosis that exists in human diabetic patients with or without retinopathy is different remains largely unknown. Here, we collected clinical information and fecal samples from 75 participants, including 25 diabetic patients without retinopathy (DM), 25 diabetic patients with retinopathy (DR), and 25 healthy controls (HC). The gut microbial composition in the three groups was analyzed using 16S ribosomal RNA (rRNA) gene sequencing. Microbial structure and composition differed in the three groups. The *α* and *β* diversities in both the DM and DR groups were reduced compared with those in the HC group. *Blautia* was the most abundant genus, especially in the DM group. In addition, increased levels of *Bifidobacterium* and *Lactobacillus* and decreased levels of *Escherichia-Shigella, Faecalibacterium*, *Eubacterium_hallii_group* and *Clostridium* genera were observed in the DM and DR groups compared with the HC group. Furthermore, a biomarker set of 25 bacterial families, which could distinguish patients in the DR group from those in the DM and HC groups was identified, with the area under the curve values ranging from 0.69 to 0.85. Of note, *Pasteurellaceae*, which was increased in DM and decreased in DR compared with HC, generated a high AUC (0.74) as an individual predictive biomarker. Moreover, 14 family biomarkers were associated with fasting blood glucose levels or diabetes, with most of them being negatively correlated. In summary, our study establishes compositional alterations of gut microbiota in DM and DR, suggesting the potential use of gut microbiota as a non-invasive biomarker for clinical and differential diagnosis, as well as identifying potential therapeutic targets of diabetic retinopathy.

## Introduction

Countless gut microbiota colonizes the human intestine. The number of bacteria in the adult gut increases to 10^14^, weighing approximately 1.13 kg, which is 10 times more than the total number of human cells (10^13^) (Shivaji, 2017). These microorganisms constitute a complex symbiotic ecosystem and are believed to interact with the host environment, thus influencing important physiological processes such as nutrient production, signaling pathway modulation, energy homeostasis, and anti-inflammatory responses (Pflughoeft and Versalovic, 2012). In recent years, disorder in gut bacterial composition and function has been associated with many human systemic diseases, including diabetes mellitus (Qin et al., 2012), obesity (Haro et al., 2016), depression (Zheng et al., 2020), Alzheimer’s disease (Wang et al., 2019), and cardiovascular disease (Battson et al., 2018). However, dysbiosis of the gut microbiome varies from person to person, showing a wide range of genomic variations that need to be elucidated.

Diabetes is one of the fastest growing health challenges of the 21^st^ century, with the number of adults living with the disease having more than tripled over the past two decades. According to the latest edition of the International Diabetes Federation (IDF) Diabetes Atlas, there are 463 million adults currently living with diabetes, and this number is estimated to reach 578 million by 2030, and 700 million by 2045 (IDF, 2019). Type 2 diabetes mellitus (T2DM) is the most common type of diabetes and is characterized by high blood glucose levels caused by the inability of the body’s cells to fully respond to insulin (IDF, 2019). T2DM is a multifactorial disease that is influenced by both environmental and genetic factors and is the leading cause of kidney failure, cardiovascular disease, and retinopathy (IDF, 2019). A 2010 study, which initially demonstrated the influence of gut microbiota on diabetes, reported the association between *Firmucutes* and *Bacteroidetes* was altered in Denmark diabetic patients compared with non-diabetic individuals (Larsen et al., 2010). Subsequent studies confirmed the function of microbiota in systemic metabolism and T2DM. Specifically, Qin et al. (2012) and Karlsson et al. (2013) undertook metagenomic sequencing in Chinese and Swedish diabetic individuals, respectively, and established that T2DM was characterized by a dysbiotic gut microbiota. Further studies indicated that microbial dysbiosis is associated with insulin resistance, abnormal lipid metabolism, and T2DM (Aron-Wisnewsky et al., 2021). Several underlying mechanisms have been demonstrated in mouse studies, including host signaling through lipopolysaccharides (LPS) derived from cell walls of gram-negative bacteria, short-chain fatty acids (SCFAs) produced by bacterial fermentation of dietary fiber, and bacterial regulation of bile acids (Allin et al., 2015).

Diabetic retinopathy is a major complication of diabetes and a leading cause of blindness and vision impairment. It can lead to vision loss due to macular edema and ischemia, as well as neovascularization of the retina and iris, such as vitreous hemorrhage, retinal detachment, and neovascular glaucoma (Cheung et al., 2010). In the USA, approximately 50% of individuals with T2DM may develop retinopathy or vision-threatening retinopathy (Kempen et al., 2004). The complications and increased prevalence of T2DM necessitate a new understanding of its biology and the development of effective approaches for its prevention and treatment. Increased understanding of the gut microbiota’s link to T2DM will provide new clinical strategies against diabetes. For example, several randomized controlled trials have indicated that standardized fecal bacterial transplantation can reduce insulin resistance and improve insulin sensitivity, halting the progression of diabetes (Udayappan et al., 2014; de Groot et al., 2021).

Since the idea of “gut–retina axis”, in which the gut microbiome modulated by diet, probiotics, or antibiotics, influences the development of retinal disease, was proposed, the significance of gut microbiome as a major modulator of eye disease has been increasingly recognized (Rowan et al., 2017; Rowan and Taylor, 2018). Gut microbiome dysbiosis is associated with many eye diseases, such as uveitis (Janowitz et al., 2019), glaucoma (Gong et al., 2020), and age-related macular degeneration (Zinkernagel et al., 2017). Furthermore, Beli et al. (Beli et al., 2018) reported that alterations in the gut microbiome *via* intermittent fasting could prevent retinopathy and prolong survival in mice, indicating that the gut flora can participate in the pathological process of diabetic retinopathy. However, it is still unclear whether differences exist in human gut microbiota between diabetic patients with and without retinopathy. Studies on gut microbial dysbiosis have identified the *Bacteroidetes* phylum using culture methods or have focused on mycobiome dysbiosis through Illumina sequencing of the ITS2 region (Moubayed et al., 2019; Jayasudha et al., 2020). Therefore, this study set out to systematically detect and identify differences in gut microbial compositions of diabetic individuals with (DR) or without (DM) retinopathy and to compare each group with healthy individuals (HC) based on 16S ribosomal RNA (rRNA) gene sequencing. The study aimed to provide a non-invasive method for diagnosis and identification of potential microbial targets that would enable further studies on the pathogenesis and therapeutic strategies for diabetic retinopathy.

## Materials and Methods

### Study Subject Recruitment and Fecal Sample Collection

A total of 50 diabetic patients and 25 healthy individuals were enrolled in this study; all of them were local (Hunan province in China) residents. Diabetic retinopathy was diagnosed based on the International Clinical Diabetic Retinopathy Disease Severity Scale (2002) (Wilkinson et al., 2003). The degree of retinopathy was determined using an ophthalmoscope, ultra-wide-field scanning laser ophthalmoscopy (UWF-SLO), and optical coherence tomography (OCT). Diabetic patients without apparent retinopathy were classified into diabetic mellitus group (DM), while those with retinopathy, were classified into diabetic retinopathy group (DR), regardless of the level of severity. Age and sex-matched healthy individuals were also recruited as a control group (HC), excluding those who had long-term usage of antibiotics, history of abdominal surgeries, and immunocompromised. None of the participants took antibiotics, probiotics, or prebiotics in the month prior to fecal sampling. Fecal samples were collected in sterile stool containers and kept cold and were transferred to the laboratory within 2 h of collection. After being sub-packaged, snap-frozen fecal samples were placed in liquid nitrogen and immediately stored at −80°C until analysis.

This study was approved by the Ethics Committee of the AIER Eye Hospital Group (Ethics No. AIER2018IRB21). The protocol in this study conformed to the Declaration of Helsinki, and all participants provided informed consent.

### DNA Extraction and Polymerase Chain Reaction Amplification

Microbial DNA was extracted from frozen fecal samples using E.Z.N.A.^®^ Soil DNA Kit (Omega Bio-tek, Inc., USA) according to the manufacturer’s protocol. DNA purity and concentration were determined using a NanoDrop 2000 UV-vis spectrophotometer, and DNA integrity was checked *via* 1% agarose gel electrophoresis. The V3 and V4 hypervariable regions of the bacterial 16S rRNA gene were chosen as targets for amplification using PCR with indexed barcodes. The primer sequences used were: 338F (5′-ACTCCTACGGGAGGCAGCAG-3′) and 806R (5′-GGACTACHVGGGTWTCTAAT-3′). PCR reactions were performed using TransStart Fastpfu DNA Polymerase (TransGen, Beijing, China) in 20 μl reaction mixtures. The amplicon products were purified using an AxyPrep DNA Gel Extraction Kit (AXYGEN Biosciences, Union City, CA, USA) and separated on 2% agarose gels. Purified amplicon products were quantified and homogenized using a Picogreen dye fluorometer and paired-end sequenced on an Illumina MiSeq platform (Illumina, San Diego, USA) according to the standard protocols of Majorbio Bio-pharm Technology Co., Ltd (Shanghai, China).

### 16S rRNA Gene Sequence and Microbiota Analysis

To obtain clean data, the raw sequence data were merged, and quality control was performed using Trimmomatic and then merged by adjusting short reads in several steps. First, reads with quality scores lower than 20 were truncated, with a minimum read length set to 50 bp. Second, overlaps longer than 10 bp were merged based on the correlation between PE reads and overlap, with a mismatch ratio of no more than 0.2. Third, samples were separated according to the barcode/primer at both ends of the sequence. The high-quality sequences were clustered into operational taxonomic units (OTUs) with 97% similarity cutoffs using Uparse v7.1 (http://drive5.com/uparse/). The taxonomy of each 16S rRNA gene sequence was analyzed using the Ribosomal Database Project (RDP) classifier v2.11 (https://sourceforge.net/projects/rdp-classifier/) based on the Silva database v132 (https://www.arb-silva.de/). The grouping information of the fecal samples was not disclosed during the processing and sequencing phases until sequence data were being analyzed.

### Statistical Analysis

Statistical analyses were performed using SPSS v25 (IBM, USA), and plots were generated using GraphPad Prism v8.0. Chi-square test was used to compare categorical variables, including gender, family history of diabetes, and so on. One-way ANOVA was used to compare continuous variables, including age, body mass index (BMI), fasting blood glucose (FBG), and duration of type 2 diabetes (abbreviated as T2D year). *α*-diversity was evaluated by the species indices (Ace, Chao, Shannon, Invsimpson, Shannoneven, and Heip), which were calculated using mother v1.30.1. *β*-diversity was used to explore diversity in microbial structure. Permutational Multivariate Analysis of Variance was used to test differences in the three groups and was performed in R studio v3.7.1 using the package “vegan”, with argument permutations = 999 and distance = “Bray–Curtis”. Linear discriminant analysis Effect Size (LEfSe) was carried out to identify the different bacterial taxa in the three groups [linear discriminant analysis (LDA) > 2.5]. The random forest classifier (R, randomForest package) was used to predict the discrimination in DM *vs.* HC, DR *vs*. HC and DM *vs*. DR. Five hundred trees were considered in each combination. The biomarkers for the random forest model evaluation were selected based on the value of the area under the curve (AUC), and the effect was verified through receiver operating characteristic (ROC) curve analysis (R, plotROC package). The two-way corrnetwork was deduced from the abundance of DM-, DR- and HC-related families using the NetworkX software v1.11, and analyzed using Spearman’s rank correlation analysis (r ≥ 0.1, *p* < 0.05). The two-way corrnetwork was deduced from selected biomarkers at the family level and two clinical parameters, using the NetworkX software, and analyzed using Spearman’s rank correlation analysis (r ≥ 0.1, *p* < 0.05). The non-parametric factorial Kruskal–Wallis sum-rank test was used to compare the relative abundances of microbes obtained from 16S rRNA sequence data using SPSS v25.0. Outcomes are presented as mean ± standard deviation (SD). The statistical significance of multiple comparisons was corrected using Bonferroni correction, and a significant correlation was set at *p* <0.05.

## Results

### Clinical Characteristics of Recruited Subjects

**Figure 1:**
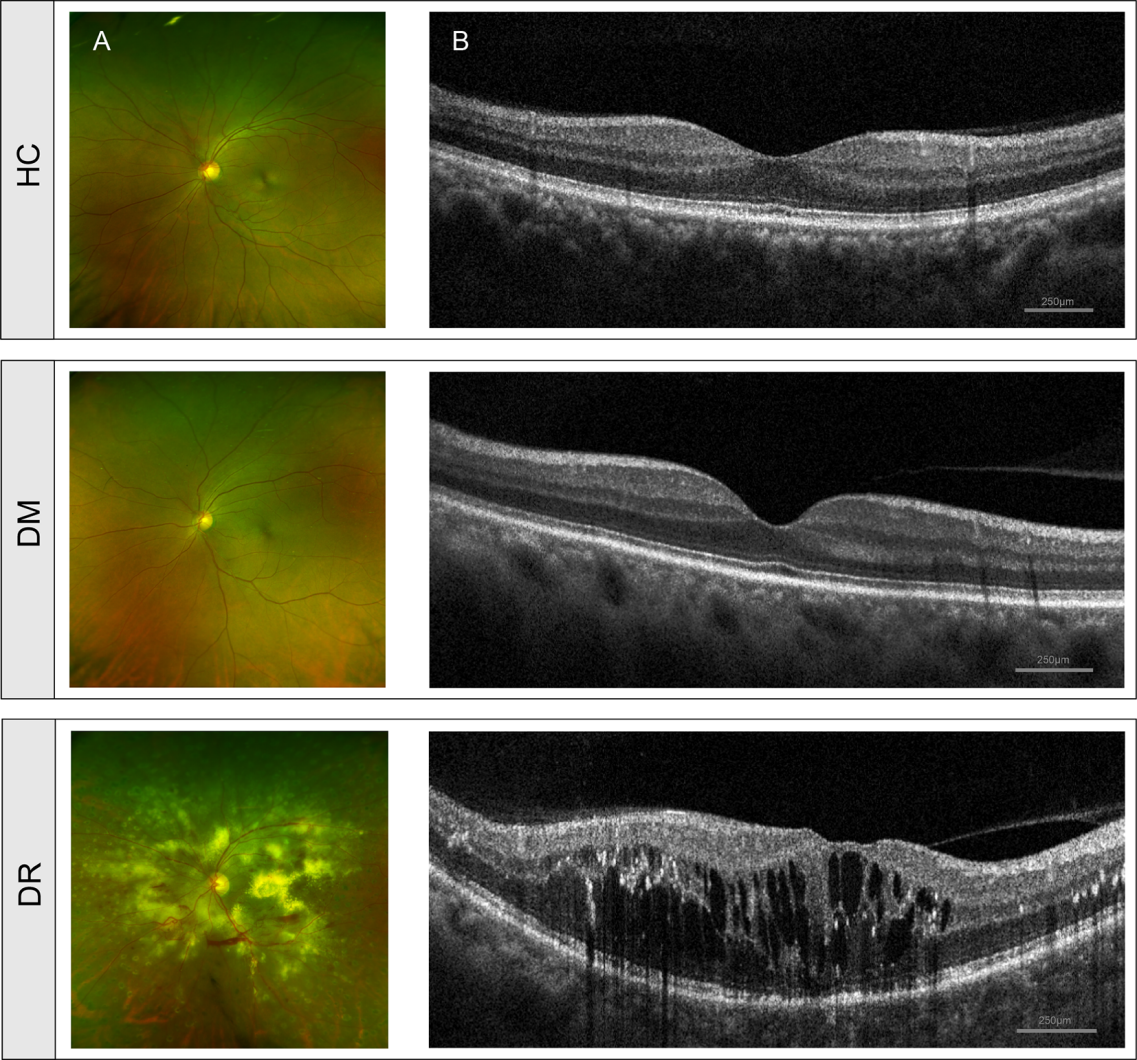
Clinical features of diabetic patients without retinopathy (DM), diabetic patients with retinopathy (DR) and healthy controls (HC). **(A)** Fundus images from ultra-wide-field fundus ophthalmoscopy showed normal fundus structure in the DM and HC groups but showed retinal vascular lesions, including microaneurysms, hemorrhages, cotton wool spots, and lipid exudates in the DR group. **(B)** OCT images showed normal histological form in DM and HC groups but showed macular edema, retinal thickening, and retinal detachment in the DR group. OCT, optical coherence tomography.

A total of 75 participants, including 50 diabetic and 25 healthy individuals were recruited into the study. Depending on the absence or presence of diabetic retinopathy, diabetic patients were divided into the diabetes mellitus without retinopathy group (DM, n = 25) or the diabetes mellitus with retinopathy group (DR, n = 25). An additional 25 age-, gender-, and BMI-matched healthy individuals were recruited into a control group (HC). Comparisons of age and BMI in the three groups did not show statistically significant differences (*p* > 0.05), while FBG and duration showed significant differences (*p* < 0.001); additional detailed characteristics (family history of diabetes, severity scale of diabetic retinopathy, medication usage, and so on) of the study subjects are included in the Supporting Information (**Supplementary Table S1**). Fundus and OCT images of individuals with DM and HC showed a normal fundus structure. In contrast, the ultra-wide-field fundus images of DR patients showed various visible retinal vascular lesions, including microaneurysms, hemorrhages, cotton wool spots, and lipid exudates (**Figure 1A**). OCT images in the DR group showed significant macular edema, retinal thickening, and retinal detachment (**Figure 1B**).

### Comparison of *α*-Diversity and *β*-Diversity in DM, DR, and HC Groups

**Figure 2:**
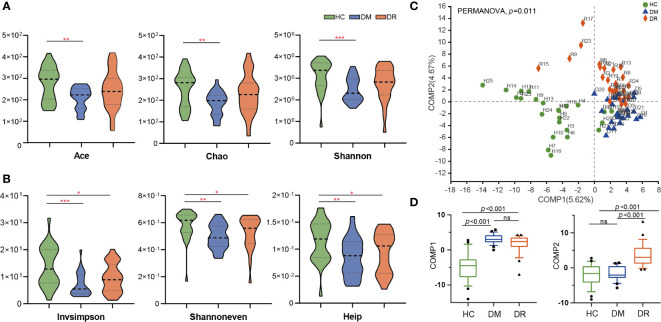
Comparison of *α*-diversity and *β*-diversity in diabetic patients without retinopathy (DM), diabetic patients with retinopathy (DR), and healthy controls (HC). **(A)** Ace, Chao, and Shannon *α*-diversity indices were significantly decreased in DM compared with HC (*p* = 0.0026, *p* = 0.0030 and *p* = 0.0005, respectively, Wilcoxon rank-sum test). However, these three indices were not statistically different between the DM and DR groups, or the DR and HC groups. **(B)** Invsimpson, Shannoneven, and Heip *α*-diversity indices were significantly decreased in the DM and DR groups relative to the HC group (*p* < 0.05). However, these indices did not reach statistical significance between the DM and DR groups. **(C)** PLS-DA analysis showed a distinct separation in the three groups at the OTU level (PERMANOVA, *p* = 0.011). **(D)** At the component 1 (COMP1) PLS-DA analysis, both DM and DR were significantly different from HC. At the COMP2 PLS-DA analysis, both HC and DM were significantly different from DR (all *p* < 0.001; Kruskal–Wallis test). PLS-DA, Partial Least Squares Discriminant Analysis. OUT, operational taxonomic units. *p < 0.05, **p < 0.01,***p < 0.001; ns, no significance.

We obtained 4,468,745 high-quality sequences across all samples, with an average length of 410.45. Based on the minimum number of sequences per sample, these sequences were filtered and clustered into 919 OTUs at 97% sequence similarity. In this study, DM and DR were strongly associated with a decrease in intra-individual diversity, as measured using *α*-diversity indices, including species richness indices (Ace and Chao), species diversity indices (Shannon and Invsimpson), and species evenness indices (Shannoneven and Heip). We found that the Ace, Chao, and Shannon indices were significantly lower in patients with DM relative to HC (*p* = 0.0026, *p* = 0.0030, and *p* = 0.0005, respectively, Wilcoxon test). However, these three indices were not statistically different between the DM and DR groups, or the DR and HC groups, although a declining trend was observed in the DR group (**Figure 2A**). At the same time, we found that the Invsimpson, Shannoneven, and Heip indices were significantly lower in the DM and DR groups relative to the HC group (*p* < 0.05); however, these indices did not reach statistical significance between the DM and DR groups (**Figure 2B**).

We performed *β*-diversity analysis to assess the overall differences and similarities in microbial population structure among the groups. Partial least squares discriminant analysis (PLS-DA) distinguished the groups at the OTU level, and permutational multivariate analysis of variance (PERMANOVA) demonstrated significant differences among them (*p* = 0.011, **Figure 2C**). The Kruskal–Wallis test was used to test the statistical significance for the two components obtained from the PLS-DA model. In component 1 (COMP1) PLS-DA analysis, both DM and DR were significantly different from HC. In COMP2 PLS-DA analysis, both HC and DM were significantly different from the DR group (all *p* < 0.001, **Figure 2D**). In addition, principal coordinate analysis (PCoA) showed that the microbial composition in the three groups was significantly different based on Bray–Curtis distances (R² = 0.048, *p* = 0.007, **Supplementary Figure S1A**), weighted UniFrac distances (R² = 0.045, *p* = 0.029, **Supplementary Figure S1B**), and unweighted UniFrac distances (R² = 0.051, *p* = 0.004, **Supplementary Figure S1C**). In summary, the above analyses showed that microbial composition of the DM and DR groups were altered relative to that of the HC group, indicating that diabetic patients show dysbiosis in gut microecology.

### Different Bacterial Composition and Abundance in DM, DR, and HC Groups

**Figure 3:**
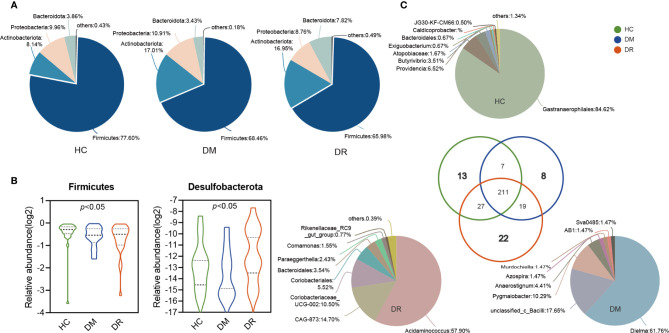
Variations in fecal microbiota composition at the genus level in diabetic patients without retinopathy (DM), diabetic patients with retinopathy (DR), and healthy controls. **(A)** The pie shows the relative proportions of bacterial phyla in the DM, DR, and controls, respectively. The *Firmicutes, Actinobacteria, Proteobacteria*, and *Bacteroidota* were the most dominant phyla in each group. *Firmicutes* levels were lower in the DR group compared with the DM and HC groups. There were no statistical differences in *Actinobacteriota*, *Proteobacteria*, and *Bacteroidota* in the three groups. **(B)** The relative abundance of *Firmicutes* and *Desulfobacterota* showed significant differences in the three groups (both *p* < 0.05; Kruskal–Wallis test). **(C)** The pie shows the overlapped and unique genera in the three groups. Most of the genera (211) overlapped and were observed in all groups. In total, 13, 8, and 22 genera were detected only in HC, DM, and DR groups, respectively.

The relative abundance of microbial composition in the three groups was compared at the phylum and genus levels. We identified 16 phyla in all the samples. *Firmutes*, *Actinobacteriota*, *Proteobacteria*, and *Bacteroidota* were the most dominant phyla in each group, accounting for more than 99% of community abundance. Overall, comparing the relative abundance of each phylum in the three groups, only *Firmicutes* and *Desulfobacterota* phyla showed significant differences (both *p* < 0.05; Kruskal–Wallis test; **Figure 3B**). *Firmutes* was less abundant in the DR group than in the DM and HC groups. There were no statistical differences in *Actinobacteriota*, *Proteobacteria*, and *Bacteroidota* in the three groups, although the trends that *Bacteroidota* was more abundant in the DR group while *Actinobacteriota* and *Proteobacteria* were higher in the DM group were observed (**Figure 3A**). Additionally, less abundant phyla that varied to different degrees were also found (**Supplementary Table S2**).

A total of 307 genera were identified. Using circos analysis, 30 major genera were distributed with different relative abundances in the three groups. Among these, *Blautia* was the most abundant genus, particularly in the DM group. Higher abundance of *Bifidobacterium* and *Lactobacillus* genera and lower abundance of *Escherichia-Shigella*, *Faecalibacterium*, *Eubacterium_hallii_group*, and Clostridium (including *Clostridium sensu_stricto_1* and *norank_f_norank_o_Clostridia_UCG-014*) genera were observed in the DM and DR groups than in the HC group. In addition, some genera, whose relative abundance was lower than 0.01, were clustered into a separate group (named others) (**Supplementary Figures S2A, B**). Most of the genera (211) overlapped and were observed in all groups. Of the 13 genera that were detected only in the HC group, 91.14% belonged to the *norank_o_Gastranaerophilales* and *Morganellaceae* families. Eight genera were detected only in the DM group, with 89.7% belonging to *Erysipelotrichaceae*, *unclassified_c_Bacilli*, and *Ruminococcaceae* families. Twenty-two genera were detected in the DR group only, and 88.62% of these belonged to *Acidaminococcaceae*, *Muribaculacea*, *Atopobiaceae*, and *norank_o_Coriobacteriales* families (**Figure 3C** and **Supplementary Table S3**).

### Distinct Gut Microbiota in DM, DR, and HC Groups

**Figure 4:**
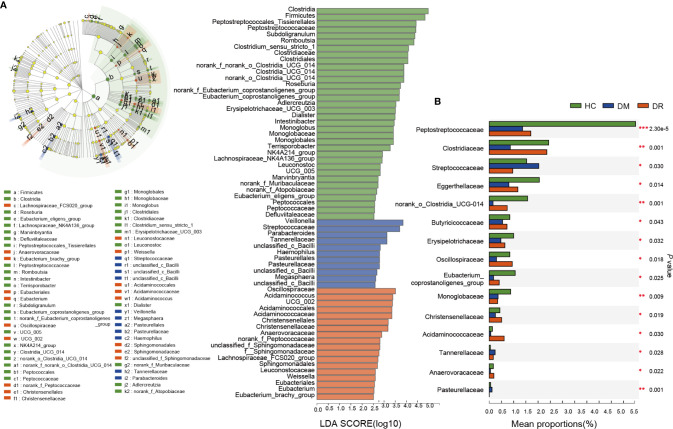
Relative abundance of the bacterial community in diabetic patients without retinopathy (DM), diabetic patients with retinopathy (DR), and healthy controls (HC). **(A)** On the left, LEfSe analysis of the fecal microbiota composition from the phylum to the genus level in the three groups. On the right, LDA effect size analysis showed the relative abundance of genera in the DM, DR, and control groups. In total, 63 bacterial taxa showed significant differences in relative abundance, with 11 and 18 distinct microbial taxa in the DM and DR groups, respectively (LDA score > 2.5, *p* < 0.05, Kruskal–Wallis test). **(B)** The top 15 significant high-abundance families; three families were enriched in the DM group; four families were enriched in the DR group; and eight families were enriched in the HC group. LEfSe, Linear discriminant analysis Effect Size; LDA, linear discriminant analysis. *p < 0.05, **p < 0.01, ***p < 0.001.

To characterize the distinct microbiota in DM, DR, and control groups, we performed LFfSe analysis on the fecal microbiota composition from the phylum to the genus level. There were 63 bacterial taxa that showed significant differences in relative abundances in the three groups, with 11 and 18 distinct microbial taxa in the DM and DR groups, respectively. These taxa belonged to 25 main families (LDA score > 2.5, *p* < 0.05, Kruskal–Wallis test; **Figure 4A**). We further analyzed significant differences at the family level using Kruskal–Wallis test to identify the bacterial family that could discriminate the DM, DR, and HC groups. The relative abundances of 26 families were significantly different. This result was similar to that of the distinct families identified using LFfSe analysis (**Supplementary Table S4**). Among the top 15 significantly different, high-abundance families, three families (*Streptococcaceae*, *Tannerellaceae*, and *Pasteurellaceae*) were significantly enriched in the DM group, while four families (*Oscillospiraceae*, *Christensenellaceae*, *Acidaminococcaceae*, and *Anaerovoracaceae*) were significantly enriched in the DR group. In contrast, eight families (*Peptostreptococcaceae*, *Clostridiaceae*, *Eggerthellaceae*, *norank_o_Clostridia_UCG-014*, *Butyricicoccaceae*, *Erysipelotrichaceae*, *Eubacterium_coprostanoligenes_group*, and *Monoglobaceae*) were significantly enriched in the HC group (**Figure 4B**).

### Gut Microbial Biomarkers for Discriminating DM, DR, and HC

**Figure 5:**
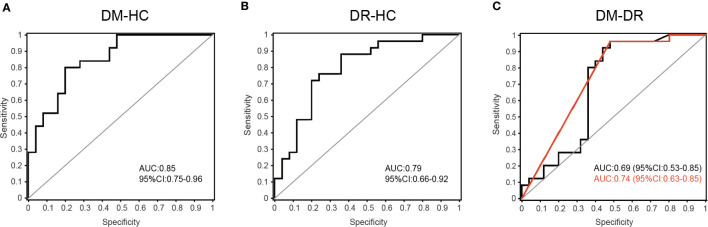
Disease classification based on gut microbial biomarkers. The classifying ability was quantified using ROC curve analysis and assessed by AUC values in **(A)** DM *versus* HC (AUC = 0.85), **(B)** DR *versus* HC (AUC = 0.79) and **(C)** DM *versus* DR (AUC = 0.69). The red line represents the result when only the *Pasteurellaceae* family was used as predictor to distinguish DM and DR groups (AUC = 0.74, 95% CI 0.63–0.85).

Bacterial families were analyzed using a random forest classifier to identify microbial signatures capable of discriminating DM, DR, and HC groups. Using model evaluation, we chose the top important features with the highest AUC values as biomarkers for distinguishing the three groups. In total, 33 important families were selected (17 for DM *vs*. HC, 10 for DR *vs*. HC and six for DM *vs*. DR; **Supplementary Figures S3A–C**). After removing duplicate categories, 25 families were identified as gut microbial biomarkers. Then, to assess the potential value of gut microbiota model as biomarkers, we used ROC curve analysis to quantify their classifying ability based on the AUC value. These microbial biomarker panels enabled us to distinguish subjects with DM from those with DR or HC with reliable diagnostic accuracy (DM *vs*. HC, AUC = 0.85; DR *vs*. HC, AUC = 0.79; DM *vs*. DR, AUC = 0.69; **Figures 5A–C**). We found that the *Pasteurellaceae*, *Oxalobacteraceae*, and *Gallionellaceae* families were the main biomarkers distinguishing DM and DR (**Supplementary Table S5**). Interestingly, using only the *Pasteurellaceae* family as a predictor generated a higher AUC of 0.74 (95% CI 0.63–0.85) (**Figure 5C**).

**Figure 6:**
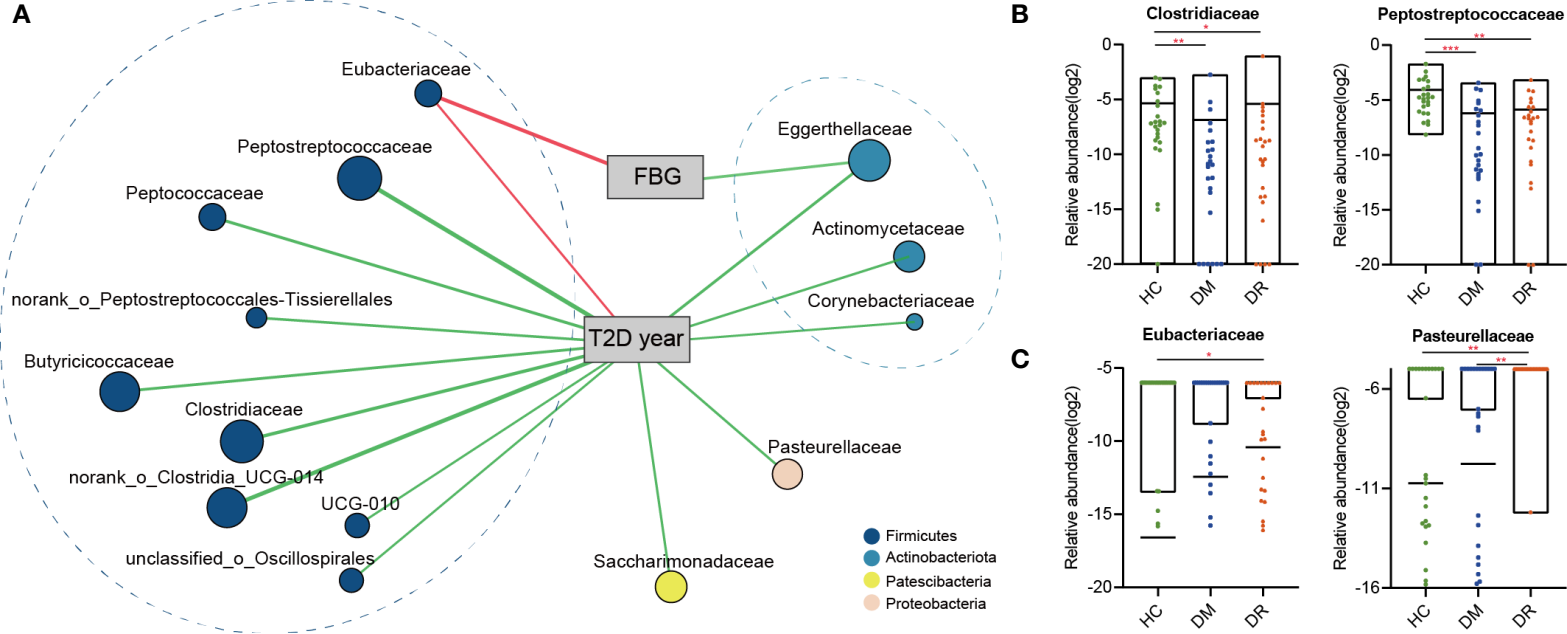
The two-way corrnetwork analysis and representative families. **(A)** The two-way corrnetwork showed Spearman correlation coefficients between the selected biomarkers and two clinical parameters; fasting blood glucose (FBG) and duration of type 2 diabetes (T2D year). Apart from *Eubacteriaceae*, which had positive correlation with FBG and duration, the other families had negative correlations. These families mainly belonged to *Firmicutes* and *Actinobacteria* phyla. **(B)** *Clostridiaceae* and *Peptostreptococcaceae* levels in DM and DR groups were lower compared with the HC group. **(C)** The highest relative abundance in *Eubacteriaceae* and the lowest relative abundance in *Pasteurellaceae* were observed in the DR group. *p < 0.05, **p < 0.01, ***p < 0.001.

To further determine whether these discriminative biomarkers could reflect diseases status, we performed Spearman’s rank correlation analysis and found that 14 families were associated with two clinical parameters: FBG and duration. Apart from *Eubacteriaceae*, which was enriched in the DR group and had positive correlation with FBG and duration, the other families showed negative correlations. These families mainly belonged to *Firmicutes* and *Actiobacteriota* phyla and most of them were enriched in the HC group (**Figure 6A** and **Supplementary Table S6**). Based on four representative families, *Clostridiaceae* and *Peptostreptococcaceae* levels were lower in the DM and DR groups than in the HC group. When we compared DM and DR groups, we found that the taxa were present at higher abundances in the DR than in the DM, although this difference did not reach statistical significance (**Figure 6B**). *Eubacteriaceae* showed the highest relative abundance while *Pasteurellaceae* showed the lowest relative abundance in the DR group compared with the DM and HC groups (**Figure 6C**). Together, we identified the bacterial biomarkers that enabled discrimination of the DM, DR, and HC groups, and some gut microbial markers reflected the glucose abnormality and duration of the disease.

## Discussion

The occurrence of diabetes and related retinopathy is closely related to gut microbial dysbiosis. Here, we applied 16S rRNA gene sequencing to obtain a comprehensive composition of gut microbiota in DM and DR patients and in healthy individuals. In addition to the unique microbial genera identified in each group, we also compared distinct genera and families of bacteria in DM and DR groups with the HC group and with each other. Furthermore, we selected and confirmed 25 biomarkers at the family level using the random forest prediction model, which could distinguish DR from DM, and both from HC, with a reliable diagnostic accuracy. Therefore, our results should be explored further in clinical trials. Moreover, the selected biomarkers can be used not only for clinical diagnosis but also as targets of novel therapeutic interventions for diabetic retinopathy.

In this study, we found that patients in the DM and DR groups had distinct gut microbiota compared with controls, based on reduced diversity and altered microbial composition. Overall, *α*-diversity indices (Invsimpson, Shannoneven, and Heip) were lower in DM and DR than in HC. Other indices (Ace, Chao, and Shannon) were lower in the DM group, but non-significantly lower in the DR group. When DR was compared with DM, an increased trend was observed in DR, although it did not reach statistical difference. DR was characterized by alterations in specific OTUs mainly assigned to the families *Acidaminococcaceae*, *Muribaculacea*, *Atopobiaceae*, and *norank_o_Coriobacteriales*. Herein, we found twenty-two genera in DR only compared with eight in DM and thirteen in HC (**Figure 3C**). We speculate that the possible explanation was the microbiota in DR might tend to exhibit a more complex and higher pathology diversity. Moreover, differences in *β*-diversity (PLS-DA) were observed in the three groups. Our results were consistent with those of previous studies, which reported that lower *α*-diversity indices (Shannon and Chao) of gut microbiota and different *β*-diversity were observed in T2DM patients compared with non-diabetic individuals (Nuli et al., 2019; Zhao et al., 2019; Reitmeier et al., 2020). Therefore, we postulate that the onset and progression of diabetes and its retinal complications are related to alterations in the gut microbiota.

We also found that DM and DR were associated with disturbance of *Firmicutes*, *Bacteroidetes*, and *Desulfobacterota* phyla. An earlier study showed that the proportions of *Firmicutes* and *Clostridia* (belonging to phylum *Firmicutes*) were significantly reduced, while that of *Bacteroidetes* was increased, in the gut of T2DM patients (Larsen et al., 2010). Similarly, we observed that abundances of *Firmicutes* and related *Clostridiaceae* in the DM and DR groups were lower than in healthy participants. Additionally, we found that *Bacteroidetes* was more abundant in the DR than in the DM and HC groups, which is in line with other study findings (Moubayed et al., 2019). *Bacteroidetes* are gram-negative bacteria whose cell walls are mainly composed of LPS. LPS derived from members of *Bacteroidetes* not only inhibit innate immune signaling and endotoxin tolerance, but are also associated with diabetic pathogenesis (Davis-Richardson et al., 2014; Vatanen et al., 2016). Vagaja et al. (Vagaja et al., 2013) reported that systemic LPS exposure in hyperglycemic mice could accelerate the injury of retinal capillary endothelium and thinning of the retina. Interestingly, we noted that, compared with the HC group, *Desulfobacterota* was enriched in the DR group but reduced in the DM group. *Desulfobacterota* is composed of many organisms that can reduce sulfur compounds *via* the DsrAB-dissimilatory sulfite reduction pathway (Waite et al., 2020), and these organisms participate in butyrate degradation by carrying out a butyrate beta-oxidation pathway, indicating that *Desulfobacterota* is involved in the equilibrium of the catabolic reaction (Hao et al., 2020). Therefore, it may be hypothesized that bacteria in the *Bacteroidetes and Desulfobacterota* phyla can either release LPS to trigger inflammatory injuries or aggravate energy metabolism abnormalities, both of which are pathological features of diabetes.

At the genus level, decreased *Faecalibacterium, Eubacterium_hallii_group*, and *Clostridium *(*Clostridium_sensu_stricto_1* and *norank_f_norank_o_Clostridia_UCG-014*) genera and increased *Blautia, Bifidobacterium*, and *Lactobacillus* were observed in both the DM and DR groups. For genera with decreased abundance, *Faecalibacterium, Eubacterium_hallii_group*, and *Clostridium* are known human gut colonizers and butyrate producers and are highly discriminant for T2DM (Karlsson et al., 2013; Hiippala et al., 2018). These bacteria ferment dietary fibers to produce butyrate through gut microbial metabolism, thus playing a regulatory role in improving insulin sensitivity, alleviating inflammation, and ameliorating diabetes in humans and mice (Vrieze et al., 2012; Udayappan et al., 2016; Hiippala et al., 2018). Similarly, reduced *Clostridium* was also found in both Chinese and European T2DM patients (Qin et al., 2012; Karlsson et al., 2013). Karlsson et al. (2013) further reported that *Clostridium* correlated negatively with fasting glucose, insulin, and plasma triglycerides, and positively with adiponectin and high-density lipoprotein, all of which are closely related to T2DM. Hence, we speculate that the occurrence of diabetes and retinopathy may be associated with reduction in these beneficial bacteria.

*Blautia* and *lactobacillus* genera were enriched in participants with diabetes and were more abundant in the DM group than in the DR group. These increased *Blautia* and *lactobacillus* genera in diabetic patients were also observed in other studies (Larsen et al., 2010; Karlsson et al., 2013; Wang et al., 2020). Wang et al. (2020) reported that *Blautia* was positively correlated with tauroursodeoxycholic acid (TUDCA) levels. TUDCA, a farnesoid X receptor (FXR) antagonist, regulates the glycolipid metabolism *via* its receptor for FXR and bile acid G-protein-coupled membrane receptor (TGR5). TGR5 can be found in retinal primary ganglion cells, and the activation of TGR5 could prevent retinopathy and prolong survival in db/db mice (Beli et al., 2018). This may explain why diabetic patients with higher *Blautia* are less likely to develop retinopathy. Increased *Lactobacilli* levels were positively correlated with fasting glucose, glycosylated hemoglobin, and a long-term measure of blood glucose control (Karlsson et al., 2013). Thus, increased *Lactobacillus* in the intestines could be a consequence of increased glucose levels in diabetic patients. However, *lactobacillus* genus represents a common probiotic with well immunomodulatory and antioxidant properties, which involved with the mechanism of diabetic retinopathy. Zeuthen et al. (Zeuthen et al., 2006) reported that lactic acid bacteria (mainly belong to *lactobacillus*) differentially up-regulated dendritic cells’ surface maturation markers and potentially contribute to the regulation of chronic inflammation. Another study revealed that oral administration of *lactobacillus* strains improved antioxidant capacity in diabetic mice (Dang et al., 2018). These beneficial effects of *lactobacillus* could partially explain the result that it was more abundant in the DM group than in the DR group. The current study found that some drugs for diabetes treatment had important effects on gut microbiome alterations. For example, Wu et al. (2017) reported that metformin significantly influenced the gut microbiome (including increased *Bifidobacterium*) to improve glucose tolerance and enhance the antidiabetic effects of the drug. Most subjects in the DM and DR groups of our study used metformin, and we noticed that *Bifidobacterium* abundance was higher in these groups than in the HC group. Therefore, the increased abundance of *Bifidobacterium* may be attributed to the effect of metformin.

Furthermore, apart from identifying the gut microbiota, microbial biomarkers should be screened that can be used to distinguish diseases. In this study, we identified 25 important families that could distinguish individuals with DR from those with DM or HC, with AUC values ranging from 0.69 to 0.85. To further develop the potential reliability of these biomarkers as diagnostic tools, we took the duration and fasting plasma glucose levels and determined their correlation with selected biomarkers. We found 14 different families, apart from *Eubacteriaceae*, which showed a positive correlation with diabetes-related clinical parameters; all the other families showed negative correlations. Most families, such as *Clostridiaceae*, *Peptostreptococcaceae*, *norank_o_Clostridia_UCG-014*, and *Butyricicoccaceae* were highly expressed in the HC group. Generally, *Clostridiaceae* and *Peptostreptococcaceae* are negatively correlated with metabolic diseases and participate in the production of SCFAs, such as acetate, propionate, and butyrate (Fei et al., 2019; Oliphant and Allen-Vercoe, 2019). Previous studies reported that SCFAs could improve glucose homeostasis and were associated with reduced risk of T2DM, obesity, and cardiovascular disease (Chambers et al., 2018; Zhao et al., 2018). This may explain the observation why their abundances were decreased in the DM and DR groups compared to controls. However, we also noted that some bacteria in DR were closer to HC in comparison with the DM group; it was partially contradictory with expectation. We presume that one possible explanation is bacterial compensatory growth. Sometimes, the abundances of SCFA-producing bacteria and the fecal SCFA concentrations are not consistent. For example, Zhao et al. (2019) reported that in most T2DM patients with diabetic complications, the abundances of SCFA-producing bacteria were increased, while the fecal SCFA concentrations, which reflect a balance status between gut production and absorption of SCFAs (Fernandes et al., 2014), were decreased. On the other hand, hyperglycemia drives intestinal barrier disruption and dysfunction of intestinal epithelial cells, which absorb and utilize SCFAs as their key energy source (Thaiss et al., 2018; Yao et al., 2020). Most of these contradictory microbiota are SCFA-producing bacteria; we presume that they might present compensatory growth in order to promote energy harvest by producing more beneficial SCFAs. Another possible reason is that the role of these bacteria was not simply to produce SCFAs. However, further research is needed, as there remains no mechanistic explanation of these microbiota toward diabetic development. In addition, we identified some microbiota, including *norank_o_Clostridia_UCG-014* and *Butyricicoccaceae*, which are worth further analysis, even though they have not been previously reported.

*Eubacteriaceae* was the only family that showed a positive correlation with fasting glucose level and was significantly enriched in diabetic patients, especially in the DR group. Generally, *Eubacteriaceae* is one of the predominant components of human gut microbiota and is abundant in healthy people (Arrieta & Finlay, 2012). However, *Eubacteriaceae* is also abundant in some immune and metabolic diseases. For example, *Eubacteriaceae* level was elevated at the time of acute mucosal simian immunodeficiency virus replication, and it was more abundant in pre-surgery, among those who went into remission from T2DM in post-surgery (Allers et al., 2020; Davies et al., 2020). The above findings indicate that *Eubacteriaceae* may play an important role in immune and metabolic diseases. Although we cannot explain the reason for the observed increase in *Eubacteriaceae* in the DM and DR groups, this observation is consistent with the relationship between *Eubacteriaceae* and metabolic diseases. Moreover, a previous study showed that *Pasteurellaceae*, which mainly colonizes the mucosal surface of the respiratory and genital tracts, is an important human and animal pathogen, and can initiate infection by employing multiple molecular mechanisms of adherence (Jacques and Paradis, 1998). Paradoxically, we found that *Pasteurellaceae* was significantly enriched in the DM group but significantly reduced in the DR group. Furthermore, using only *Pasteurellaceae* as a biomarker to distinguish between the DM and DR groups reached the highest accuracy (AUC: 0.74). We propose that *Pasteurellaceae* might be the major microbiota, and its effects on DM and DR requires further studies involving larger sample sizes.

The main strength of this study is that the recruited subjects were strictly control-matched, with well-characterized clinical information, thus reducing bias arising from potential confounding effects such as age, sex, and BMI. In addition, previous studies have shown that the gut microbial composition may be influenced by antibiotics (Mikkelsen et al., 2015; Ianiro et al., 2016). We recruited subjects with no recent history of antibiotic use. However, there were some limitations to our study. For example, there is a certain admission-rate bias because a large proportion of subjects were selected in the hospital. In addition, owing to lack of detailed dietary information for the subjects, our study could not control for or assess whether and how dietary habits influenced gut microbial composition in the DM, DR, or HC groups. As Hunan province is located in southern China, and rice is the predominantly food here, whether the dietary habits of different regions have any effects on the microbial changes will require further multicenter controlled studies. Furthermore, our findings do not show a causal relationship between the identified differential gut microbial composition in each group, which is an inherent limitation of cross-sectional studies. To extend and advance the current work, a larger sample cohort and other emerging analytical strategies need to be considered in future studies.

## Conclusion

We have characterized and identified distinct gut microbial compositions in subjects in the DR and DM groups and compared them with healthy controls. Moreover, we have developed and validated a gut microbial classifier model that can effectively discriminate the DR from the DM and HC groups. The current findings lay the foundations for future studies, based on distinct gut microbiota, for developing better clinical diagnostic methods and identifying potential therapeutic targets of diabetic retinopathy.

## Data Availability Statement

The datasets presented in this study can be found in online repositories. The names of the repository/repositories and accession number(s) can be found below: NCBI BioProject, accession no: PRJNA688998.

## Ethics Statement

The studies involving human participants were reviewed and approved by the Ethics Committee of the AIER Eye Hospital Group (Ethics No. AIER2018IRB21). The patients/participants provided their written informed consent to participate in this study. Written informed consent was obtained from the individual(s) for the publication of any potentially identifiable images or data included in this article.

## Author Contributions

YH was involved in the conception and design of the study. YH, ZW, HM, SJ, ZCh, and ZCu were involved in the collection and assembly of data. YH, JC, and ST were involved in interpreting the data and wrote the manuscript. All authors contributed to the article and approved the submitted version.

## Funding

This work was supported by the National Natural Science Foundation of China (81871495) and the Science and technology project of Changsha, Hunan (kh1901251), Key Research and Development Program of Jiangxi Province (20203BBGL73193), and Science Research Foundation of Aier Eye Hospital Group (AM2001D1).

## Conflict of Interest

The authors declare that the research was conducted in the absence of any commercial or financial relationships that could be construed as a potential conflict of interest.

## Supplementary Material

Supplementary Figure 1 Comparisons of β-diversity with PCoA in DM, DR, and healthy controls. **(A)** PCoA of Bray-curtis distances (R²=0.048, p=0.007). **(B)** PCoA of weighted UniFrac distances (R²=0.045, p=0.029). **(C)** PCoA of unweighted UniFrac distances (R²=0.051, p=0.004). Abbreviation: PCoA, principal coordinate analysis.

Supplementary Figure 2 Variations in fecal microbiota composition in DM, DR, and HC groups. **(A)** Circos represent the groups and bacterial genera. The right side shows bacterial genera, and the left side shows the three study groups. The innermost lines indicate the connections between the bacteria and group. The thickness of the lines indicates the abundance of the bacteria. The different colors represent groups and different genera. The genera whose relative abundances were lower than 0.01 were clustered into a separate group (named others). **(B)** The plots show the differences in representative genera in the three groups.

Supplementary Figure 3 Biomarkers chosen based on Random Forest classifier analysis. The top important features with the highest AUC values were chosen as biomarkers to distinguish the three groups. **(A)** Seventeen important families were selected for DM versus HC. **(B)** Ten important families were selected for DR versus HC. **(C)** Six important families were selected for DM versus DR.
