# Supplementary figures and images for: Dysbiosis and Implication of the Gut Microbiota in Diabetic Retinopathy
Source: Front Cell Infect Microbiol. 2021 Mar 19;11:646348. doi: 10.3389/fcimb.2021.646348 (PMC8017229; doi:10.3389/fcimb.2021.646348)

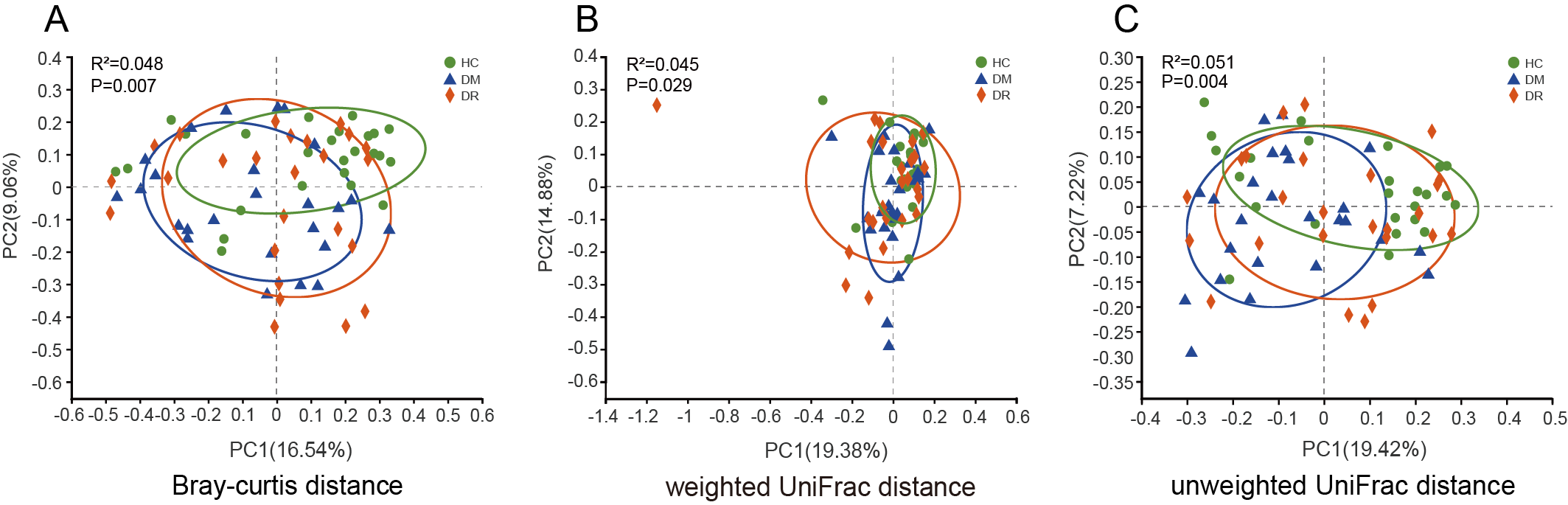

Supplement: Supplementary Figure 1 — Comparisons of β-diversity with PCoA in DM, DR, and healthy controls. (A) PCoA of Bray-curtis distances (R²=0.048, p=0.007). (B) PCoA of weighted UniFrac distances (R²=0.045, p=0.029). (C) PCoA of unweighted UniFrac distances (R²=0.051, p=0.004). Abbreviation: PCoA, principal coordinate analysis. [file Image_1.tif]

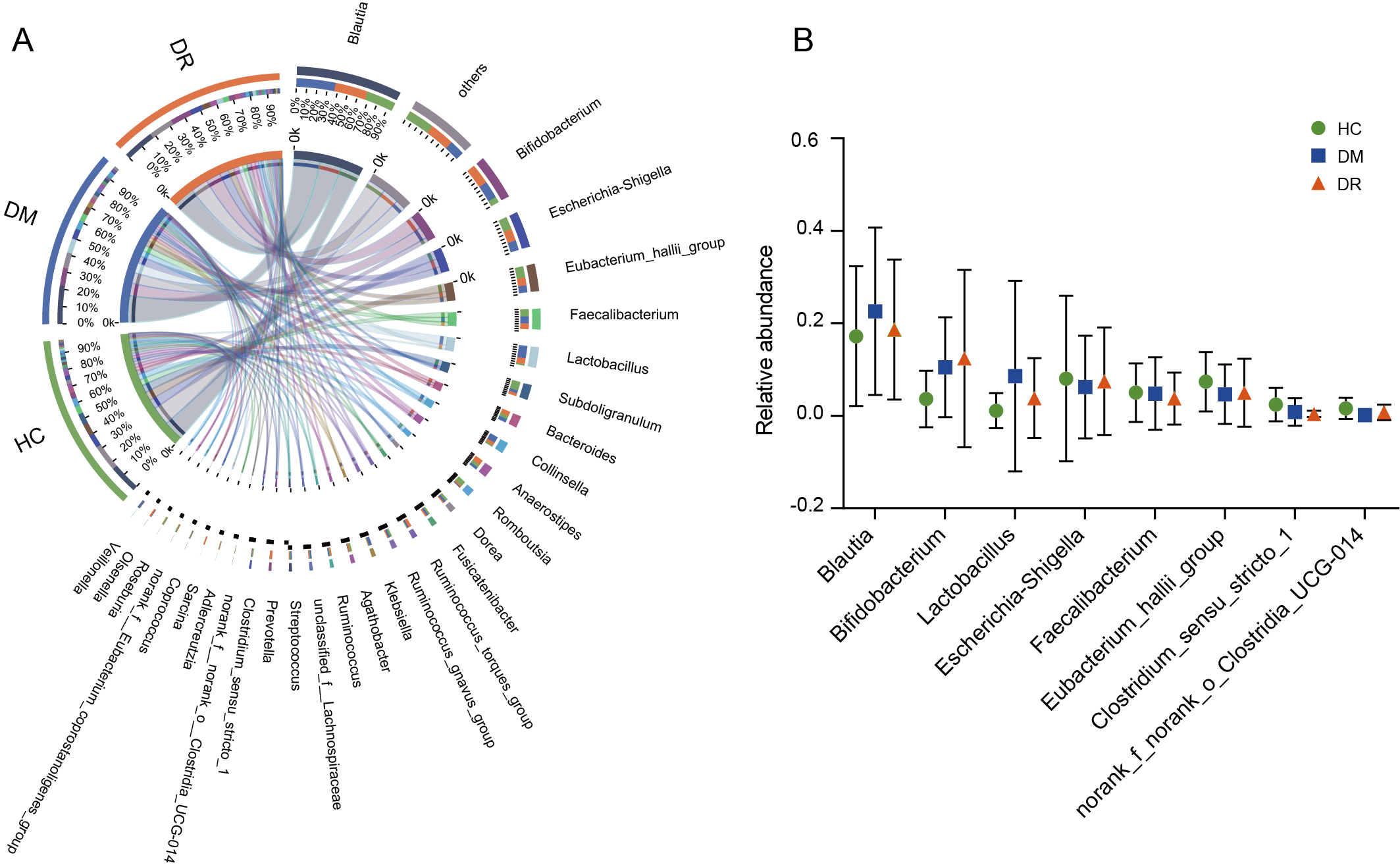

Supplement: Supplementary Figure 2 — Variations in fecal microbiota composition in DM, DR, and HC groups. (A) Circos represent the groups and bacterial genera. The right side shows bacterial genera, and the left side shows the three study groups. The innermost lines indicate the connections between the bacteria and group. The thickness of the lines indicates the abundance of the bacteria. The different colors represent groups and different genera. The genera whose relative abundances were lower than 0.01 were clustered into a separate group (named others). (B) The plots show the differences in representative genera in the three groups. [file Image_2.tif]

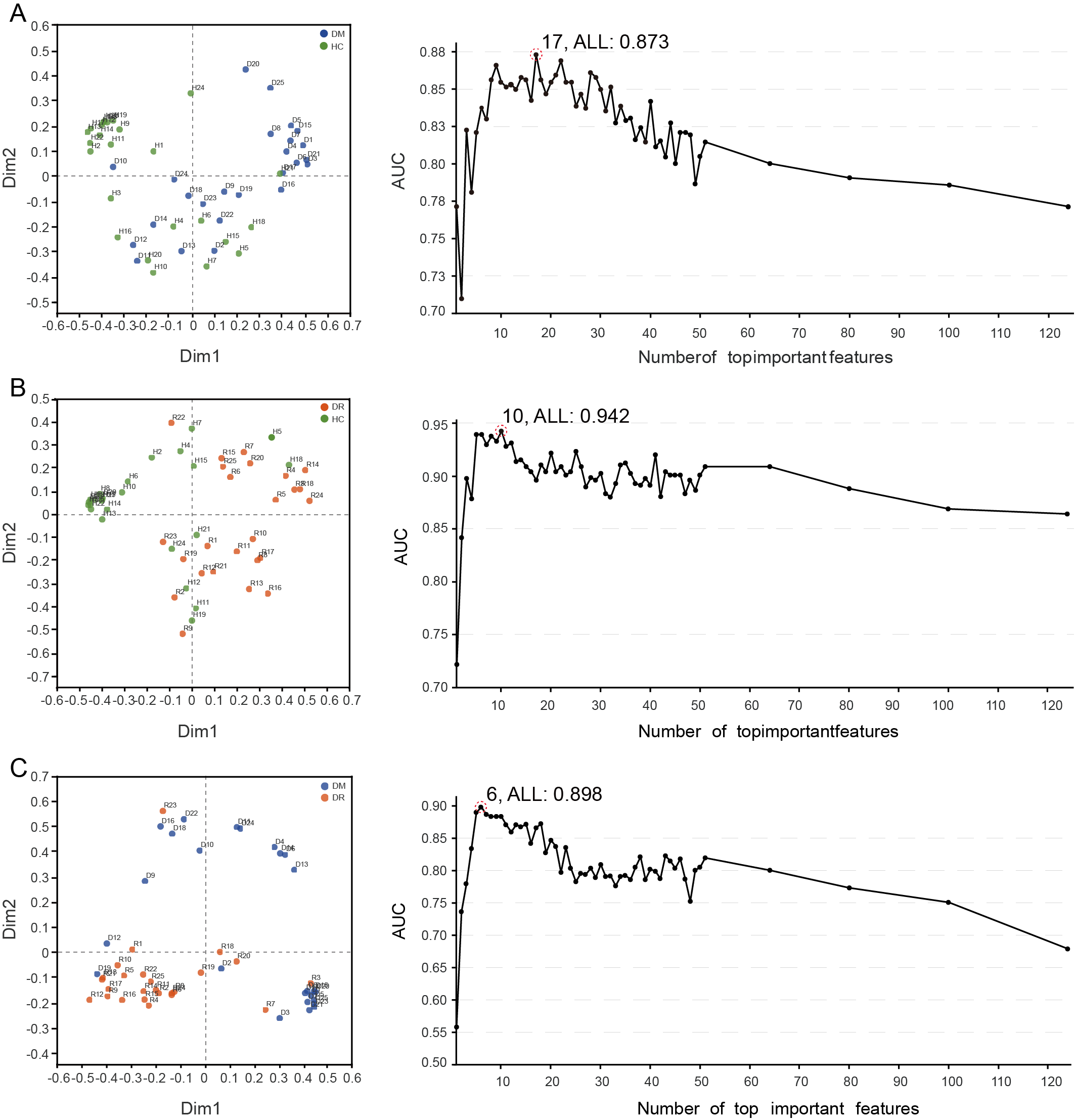

Supplement: Supplementary Figure 3 — Biomarkers chosen based on Random Forest classifier analysis. The top important features with the highest AUC values were chosen as biomarkers to distinguish the three groups. (A) Seventeen important families were selected for DM versus HC. (B) Ten important families were selected for DR versus HC. (C) Six important families were selected for DM versus DR. [file Image_3.tif]
